# Preclinical evidence to support repurposing everolimus for craving reduction during protracted drug withdrawal

**DOI:** 10.1038/s41386-021-01064-9

**Published:** 2021-06-29

**Authors:** Alvin S. Chiu, Matthew C. Kang, Laura L. Huerta Sanchez, Anne M. Fabella, Kalysta N. Holder, Brooke D. Barger, Kristina N. Elias, Christina B. Shin, C. Leonardo Jimenez Chavez, Tod E. Kippin, Karen K. Szumlinski

**Affiliations:** 1 Department of Psychological and Brain Sciences, University of California Santa Barbara, Santa Barbara, CA USA; 2 Department of Molecular, Developmental and Cell Biology and the Neuroscience Research Institute, University of California Santa Barbara, Santa Barbara, CA USA; 3 Institute for Collaborative Biotechnologies, University of California Santa Barbara, Santa Barbara, CA USA

**Keywords:** Preclinical research, Addiction

## Abstract

Cue-elicited drug-craving is a cardinal feature of addiction that intensifies (incubates) during protracted withdrawal. In a rat model, these addiction-related behavioral pathologies are mediated, respectively, by time-dependent increases in PI3K/Akt1 signaling and reduced Group 1 metabotropic glutamate receptor (mGlu) expression, within the ventromedial prefrontal cortex (vmPFC). Herein, we examined the capacity of single oral dosing with everolimus, an FDA-approved inhibitor of the PI3K/Akt effector mTOR, to reduce incubated cocaine-craving and reverse incubation-associated changes in vmPFC kinase activity and mGlu expression. Rats were trained to lever-press for intravenous infusions of cocaine or delivery of sucrose pellets and then subjected to tests for cue-reinforced responding during early (3 days) or late (30–46 days) withdrawal. Rats were gavage-infused with everolimus (0–1.0 mg/kg), either prior to testing to examine for effects upon reinforcer-seeking behavior, or immediately following testing to probe effects upon the consolidation of extinction learning. Single oral dosing with everolimus dose-dependently blocked cocaine-seeking during late withdrawal and the effect lasted at least 24 h. No everolimus effects were observed for cue-elicited sucrose-seeking or cocaine-seeking in early withdrawal. In addition, everolimus treatment, following initial cue-testing, reduced subsequent cue hyper-responsivity exhibited observed during late withdrawal, arguing a facilitation of extinction memory consolidation. everolimus’ “anti-incubation” effect was associated with a reversal of withdrawal-induced changes in indices of PI3K/Akt1/mTOR activity, as well as Homer protein and mGlu1/5 expression, within the prelimbic (PL) subregion of the prefrontal cortex. Our results indicate mTOR inhibition as a viable strategy for interrupting heightened cocaine-craving and facilitating addiction recovery during protracted withdrawal.

## Introduction

Drug-craving is a cardinal feature of addiction that can be elicited by re-exposure to drug-associated cues. Insidiously, the intensity of cue-elicited drug-craving both incubates (i.e., intensifies) during protracted withdrawal [1, 2] and becomes resistant to extinction [3–5]. These phenomena are theorized to contribute substantially to the chronic, relapsing, nature of addiction by driving perseverative cue hyper-reactivity in drug-abstinent individuals [6, 7]. The functional neuroanatomy underpinning the incubation of cue-elicited cocaine-craving involves neuroadaptations within glutamatergic corticostriatal projections, particularly those from the prelimbic cortex (PL) subregion of the ventromedial prefrontal cortex (vmPFC) to the core subregion of the nucleus accumbens (NAc) [3–10]. In humans with Cocaine use disorder, the vmPFC exhibits a time-dependent hyperactivity in response to drug-associated stimuli (e.g., [11, 12]), and interrogation of the vmPFC and NAc in rodent models of incubated cocaine-craving highlights anomalies in Group 1 metabotropic (mGlu) and calcium-permeable AMPA-type glutamate receptor expression which may reflect, for instance, deregulated mammalian target of rapamycin (mTOR) function [13, 14], as one driver of this phenomenon.

Indeed, mTOR-related signaling has received considerable experimental attention in humans with Cocaine user disorder [15] and in animal models of addiction (c.f., [16]), including incubated cocaine-craving [14] due to its ability to critically regulate intracellular signaling networks. mTOR activity is regulated up-stream by phosphoinositide 3-kinase (PI3K) and Akt1 (a.k.a. protein kinase B) [17, 18]. In addition, activated PI3K recruits Akt1 to the plasma membrane, where it can be phosphorylated at Ser473 by mTOR [18–20], among other kinases (e.g., [21]). Recently, we identified increased PI3K/Akt1 signaling within vmPFC as a biochemical correlate of incubated cocaine-craving in rats that is necessary for the expression of this behavioral phenomenon [4]. Of potential relevance to anti-craving medications development, there exists a number of FDA-approved, orally bioavailable, medications that inhibit the PI3K/Akt1/mTOR signaling pathway (c.f., [22, 23]). This fact led us to examine the effects of acute, oral, pretreatment with the FDA-approved and commercially available allosteric mTOR inhibitor everolimus (formerly RAD 001; a.k.a., Zortress, Certican, Afinitor, Votubia, Evertor) upon the incubation of cue-elicited cocaine-craving in a rat model of addiction and its relation to the activational state of PI3K/Akt1/mTOR signaling within vmPFC subregions. Rats exhibiting incubated responding also exhibit persistently high drug-seeking across days [3–5], which may reflect extinction failure and relates to a deficit in vmPFC Group 1 mGlu function [3]. Thus, the present study also examined the impact of everolimus upon the consolidation of extinction learning. Lastly, relation between the behavioral effects of inhibitor pretreatment and changes in the expression of Group 1 mGlu receptors and their associated Homer scaffolding proteins within vmPFC subregions were also determined.

Our results show that acute, oral, pretreatment with everolimus blocks incubated cocaine-craving in rats over days and reverses incubation-related protein adaptations within vmPFC subregions. Such findings demonstrate the therapeutic potential of systemic treatment with current FDA-approved PI3K/Akt1/mTOR inhibitors for reversing vmPFC neuroadaptations driving perseverative cocaine-craving.

## Materials and methods

### Subjects

Subjects were adult, male, Sprague–Dawley rats (275–325 g; Charles River Laboratories, Hollister, CA). Details of housing and animal care are summarized in the Supplementary information. Experimental protocols, as well as housing and animal care, were consistent with the guidelines provided by the National Institute of Health *Guide for Care and Use of Laboratory Animals*. The Institutional Animal Care and Use Committee of the University of California, Santa Barbara, approved all experiments.

### Surgical procedures, cocaine, and sucrose self-administration

The surgical procedures to implant jugular catheters were identical to those described previously [3–5, 9, 24–26] and are detailed in the Supplementary information. No food restriction was employed during self-administration training. Thus, rats were first trained to lever-press for their respective reinforcer during a 6-h session to increase the probability of learning. To prevent overdose, the number of cocaine reinforcers earned was capped at 102 infusions during this 6-h session, with no cap on the number of sucrose pellets earned. For the next 9 days, rats continued to self-administer their respective reinforcer during daily 2-h sessions. While our prior studies of incubated cocaine-craving employed a 10-day, 6 h/day, self-administration procedure [3–5, 9, 24, 26], incubated cocaine-craving can be elicited under shorter-access procedures [4–6]. Thus, we rationalized that if increased mTOR/PI3K/Akt1 signaling within vmPFC subregions drives incubated responding, then [1] positive correlations should be detected between cue-elicited responding and our proteins indices of mTOR/PI3K/Akt1 signaling within vmPFC subregions, irrespective of their cocaine-access [2]; rats exhibiting incubated responding under our hybrid procedures should exhibit changes in protein expression consistent with those reported for rats exhibiting incubated responding following 6 h-access procedures and [3] mTOR inhibition should reduce the incubation of cocaine-craving, regardless of the duration of cocaine-access. Additional details regarding the cocaine and sucrose self-administration procedures are provided in the Supplementary information.

### Gavage infusion procedures and cue testing

**Fig. 1:**
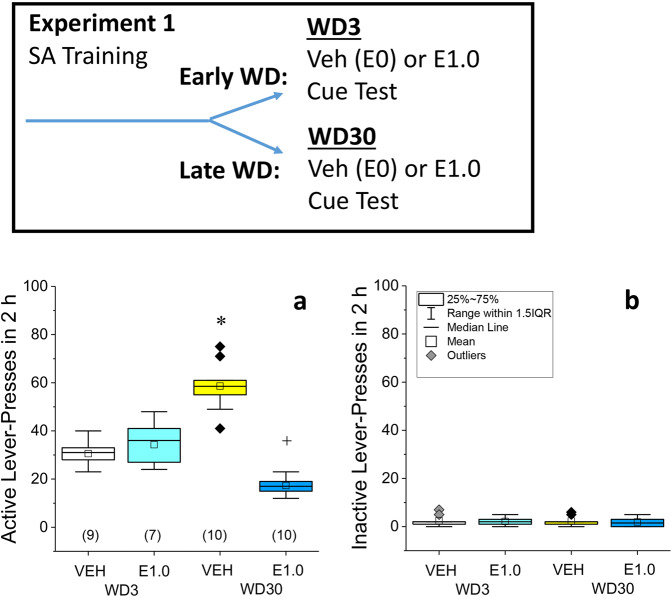
Acute everolimus pre treatment selectively blocks incubated cocaine-seeking. Inset: Summary of the procedural timeline of experiment 1. **a** Vehicle (VEH)-pretreated rats exhibited increased active lever-pressing on withdrawal day (WD) 30 vs. WD3 and this incubated responding was reduced by pretreatment with 1.0 mg/kg everolimus (E1.0). **b** No everolimus effects were observed for inactive presses during cue-testing. As summarized in **b**, the data are presented as box plots in which the mean is represented by, the median by —, outliers are indicated by ♦’s, the box represents the interquartile range (IQR) and the error bars represent 1.5 X IQR. The sample sizes are indicated in parentheses in **a**. *p < 0.05 WD30-VEH vs. WD3-VEH (incubation); ^+^*p* < 0.05 WD30-E1.0 vs. WD30-VEH (Everolimus effect) as determined by Tukey–Kramer tests, corrected for multiple comparisons.

Details of the gavage infusion procedures are provided in the Supplementary information. Experiment 1 determined the impact of oral dosing with everolimus on cocaine-seeking during early withdrawal vs. late (i.e., incubated) withdrawal (see Fig. 1, top). For this, rats were gavage-infused with vehicle (VEH; 1% DMSO in water; vol: 1 ml/kg) or everolimus (1.0 mg/kg in VEH) on withdrawal day 3 (WD3) or withdrawal day 30 (WD30). This everolimus dose is either at, or below, those demonstrated to exert therapeutic effects in other rodent models of neurological disease [22, 23, 27–36]. Testing for cocaine-seeking occurred 30 min post gavage, was conducted in the same operant chamber as that employed during the self-administration phase of the study and was 2-h long, as in prior immunoblotting work [1]. During these cue tests, depression of the formerly reinforced, “active”, lever resulted in the presentation of the 20-s tone/light stimulus, while depression of the inactive lever had no programmed consequences.

**Fig. 2:**
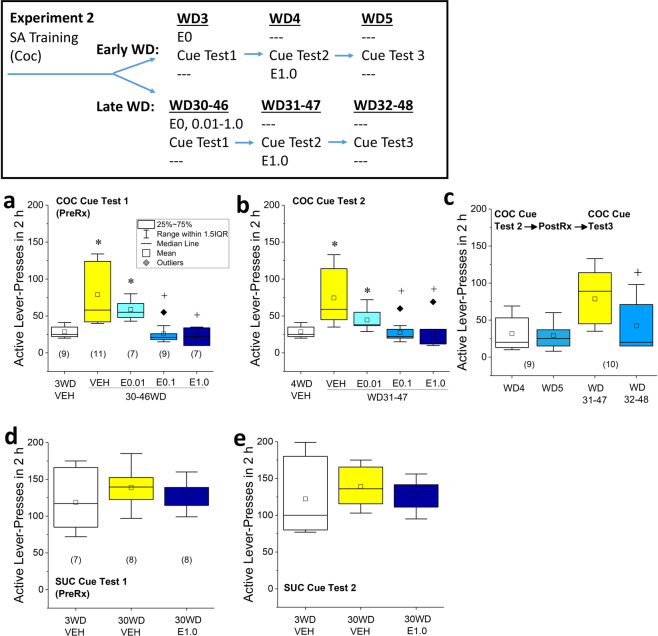
Acute everolimus pre treatment blocks the incubation of cocaine-craving. Inset: Summary of the procedural timeline of experiment 2. **a** Acute everolimus (E) pre treatment (PreRx) dose-dependently reduced incubated cue-elicited responding on the active lever, relative to vehicle (VEH)-pretreated rats and **b** this effect persisted the next day on Cue Test 2. For **a**, **b**, *p < 0.05 vs. 3WD-VEH control (incubation); ^+^*p* < 0.05 vs. 30WD-VEH control (Everolimus effect) as determined by LSD post hoc tests. **c** Pairwise comparisons of the active lever-pressing behavior between Test 2 (following which all rats were injected with 1.0 mg/kg everolimus) and Test 3 (conducted the following day) confirmed no change in responding for rats tested in early withdrawal (WD4 vs. WD5), while everolimus post treatment significantly reduced responding from Test 2 to Test 3 in incubated rats (WD31–47 vs. WD32–48). For **c**, ^+^*p* < 0.05 vs. WD31–47 as determined by a pairwise comparison (Everolimus posttreatment effect). **d** In rats trained to self-administer sucrose, cue-elicited sucrose-seeking was unaffected by withdrawal or 1.0 mg/kg everolimus on Cue Test 1 or **e** Cue Test 2 (no further treatment). As summarized in **a**, the data are presented as box plots in which the mean is represented by, the median by —, outliers are indicated by ♦’s, the box represents the interquartile range (IQR) and the error bars represent 1.5 X IQR. The sample sizes are indicated in parentheses in their respective panels.

Experiment 2 first determined the dose-response function for everolimus’ inhibition of incubated cocaine-seeking. The initial half-life of everolimus administered either PO or IV is ~12–17 h, with a terminal half-life of 49–52 h [32]. As its terminal half-life raised the possibility for carry-over effects, all rats in experiment 2 underwent were subjected to a minimum of two tests, spaced 24 h apart. At WD30–46 from cocaine self-administration, rats were pretreated with VEH or a range of everolimus doses (0.01, 0.1, and 1.0 mg/kg) at 30 min prior to testing (Cue Test 1). A group of VEH-treated rats was also tested at WD3 to provide a baseline of cue-elicited responding for ascertainment of incubation, as in prior work [5, 6] (see Fig. 2, top; Cue Test 1 conducted on WD3 vs. WD30–46). Next, rats underwent a second cue-test session (Cue Test 2) 24 h after Test 1 with no experimental manipulation between tests (see Fig. 2, top; Cue Test 1 vs. Cue Test 2, conducted on WD4 vs. WD31–47). Lastly, we examined the possibility that 1.0 mg/kg everolimus might facilitate the consolidation of extinction learning. Having established that incubated responding persisted For this, all rats that originally received VEH pretreatment prior to Cue Test 1 on WD30–46, were injected with 1.0 mg/kg everolimus immediately after Cue Test 2 (conducted on WD31–487). Then, these rats were tested a third time the following day on WD32–48 (next day (Cue Test 3)) and their behavior compared to that exhibited on Cue Test 2, using a within-subjects design. A schematic of the procedural timeline for experiment 2 is provided in Fig. 2.

Experiment 3 examined off-target effects by assessing the potential impact of everolimus (1.0 mg/kg) upon cue-reinforced sucrose-seeking during late withdrawal [9]. As everolimus did not affect cocaine-seeking behavior on WD3, its influence on sucrose-seeking at the earlier timepoint was not examined. In addition, rats were re-tested at 24 h post treatment to assess potential carry-over or delayed effects.

### Immunoblotting

To relate the “anti-incubated craving” effects of everolimus to the activational state of PI3K/Akt1/mTOR signaling and changes in mGlu/Homer expression within vmPFC subregions, we collected tissue from the rats upon the completion of experiment 1. Immediately following the cue-elicited cocaine-seeking test, rats were euthanized by rapid decapitation and the PL and IL were dissected out over ice (see Supplementary Fig. 2). The procedures for preparing tissue homogenates, detecting and quantifying the expression of [1] both the monomer and the active dimer forms of mGlu1 and mGlu5 [2]; both Homer1/c and Homer2a/b isoforms; and [3] both the phosphorylated and non-phosphorylated forms of Akt1, P70S6 kinase (P70S6K) and its major downstream effector riboprotein S6 (rpS6) [18, 37] were similar to those employed previously by our group [3, 4, 25, 38–40] and are detailed in the Supplementary information. As conducted in prior work, calnexin expression was employed to control for protein loading and transfer (see Supplementary information for details and Supplementary Fig. 3 for representative immunoblots).

### Statistical analyses

The data from the single everolimus dose study (Experiment 1) were analyzed separately at each withdrawal time-point group using Tukey–Kramer multiple comparison tests between VEH and everolimus-pretreated groups. Between-group (WD3 vs. WD30) comparisons of behavior and protein expression were also conducted in the VEH-treated rats to confirm the presence/absence of an incubated response. As these multiple comparison tests involved three comparisons, alpha was set at 0.016. Pearson’s correlational analyses were also conducted to determine the relationship between drug-seeking behavior during experiment 1 and protein expression within vmPFC subregions. Given the unbalanced design of experiment 2, the data were analyzed using mixed-design ANOVA, with Cue Test as a within-subjects factor, followed by LSD post hoc tests along the Drug factor. As the initial analyses indicated no Cue Test effect (see “Results” section), the data were collapsed across test day for post hoc comparisons using LSD tests. The data for sucrose-seeking were analyzed using a Drug X Withdrawal ANOVA. For all ANOVAs, alpha was set at 0.05. Throughout, the data are presented graphically as box plots to illustrate the data mean and median, as well as the interquartile range (IQR), with the error bars representing 1.5 X IQR. Datapoints that lie outside the 1.5 X IQR are also indicated in each figure panel.

## Results

### Everolimus pre treatment decreases cocaine-seeking only following incubation

Following 10 days of cocaine self-administration (6 h on day 1; 2 h on days 2–10), rats were pretreated with VEH or 1.0 mg/kg everolimus (E1.0) prior to a cue-elicited cocaine-seeking test on either WD3 or WD30. Prior to everolimus treatment, no group differences were apparent for the average active- or inactive-lever presses or for the number of cocaine reinforcers earned (Supplementary Table 1). VEH-pretreated rats emitted more cue-reinforced lever-presses on WD30 vs. WD3 (Fig. 1a; *p* < 0.0001), indicative of incubated craving. As depicted in Fig. 1a, everolimus blocked cue-elicited responding in rats tested on WD30 (VEH vs. E1.0, *p* < 0.0001), with no effect detected on WD3 (VEH vs. E1.0, *p* = 0.29). In contrast, inactive lever-pressing did not vary as a function of withdrawal in VEH-pretreated rats (Fig. 1b; WD3 vs. WD30, *p* = 0.88) and everolimus did not affect inactive lever-pressing on either WD3 or 30, relative to that exhibited by VEH-pretreated controls (for WD3, *p* = 0.76; for WD30, *p* = 0.63). From this single-dose study, we concluded that everolimus abolishes incubated cocaine-seeking, without altering cue-elicited cocaine-seeking during short-term withdrawal.

### Everolimus pre treatment dose-dependently blocks incubated cocaine-seeking

Consequently, we determined the dose-response function for everolimus suppression of incubated cocaine-seeking in late withdrawal. All groups exhibited comparable lever-responding and cocaine intake history prior to testing (Supplementary Table 1).

Acute everolimus pre treatment dose-dependently reduced incubated cue-elicited responding (Fig. 2a; drug effect: *F*_4,38_ = 11.14, *p* < 0.0001) and this effect persisted, unchanged, for at least 24 h (Fig. 2a vs. Fig. 2b; test effect and interaction: *F*_4,38_ < 1.0, *p*’s > 0.09). Given the persistence of responding, the data were collapsed across cue tests for LSD post hoc analyses. The VEH rats tested in late withdrawal exhibited significantly greater responding than those tested in early withdrawal (*p* < 0.0001), indicating the incubation of responding. Relative to VEH controls tested in late withdrawal, everolimus significantly lowered incubated lever-responding at all doses (for 0.01 mg/kg, *p* = 0.02; for 0.1 mg/kg, *p* < 0.0001; for 1.0 mg/kg, *p* < 0.0001). Further, the responding exhibited by the rats pretreated with the two higher everolimus doses was not different from that observed in VEH controls tested in early withdrawal (for 0.1 mg/kg, *p* = 0.54; for 1.0 mg/kg, *p* = 0.32), indicating a block of incubated cocaine-craving at these doses. The everolimus effect was selective for active lever-responding as no group differences were detected for inactive lever-pressing on either cue test (Supplementary Fig. 1a, b; Drug X Test ANOVA: *F*_4,38_ < 0.25, all *p*’s > 0.40).

### Everolimus post treatment may facilitate the consolidation of extinction learning

Next, we probed whether treating rats with 1.0 mg/kg everolimus immediately following Cue Test 2 might facilitate the consolidation of extinction learning (see Fig. 2, top). An analysis of the active lever-pressing behavior between Test 2 (post treatment) and Test 3 (no further treatment) indicated a main Test effect (*F*_1,16_ = 5.93, *p* = 0.03). Although the ANOVA failed to detect a significant main effect of, or interaction with, everolimus post treatment (*p*’s > 0.18), inspection of Fig. 2c suggested that the main Test effect was driven primarily by the rats tested in late withdrawal—an observation supported by a direct comparison of these data (*t*_9_ = 2.49, *p* = 0.04). No effect of everolimus post treatment was observed on inactive lever-pressing between Cue Test 2 and 3 (Supplementary Fig. 1c; *p*’s > 0.12). These latter data argue that the “anti-incubation” effect of everolimus may involve a facilitation of extinction memory consolidation.

### Everolimus pre treatment does not alter sucrose-seeking

Finally, we determined the impact of everolimus on sucrose-seeking in late withdrawal (i.e., WD30). No group differences in operant responding or reinforcement were noted prior to treatment with 1.0 mg/kg everolimus (Supplementary Table 1). We detected no evidence of incubated sucrose-seeking in ad libitum-fed and -watered rats or effects of 1.0 mg/kg everolimus upon cue-reinforced responding (Fig. 2e, f; Drug X Test ANOVA: *F*_1,20_ or *F*_2,20_ < 0.70, all *p*’s > 0.50) or inactive lever-pressing (Supplementary Fig. 1d, e; Test effect: *F*_1,20_ = 6.00, *p* = 0.02; drug effect and interaction: *F*_1,20_ and *F*_2,20_ < 4.0, *p*’s > 0.07). Thus, acute pretreatment with 1.0 mg/kg everolimus does not impact cue-elicited sucrose-seeking.

### Everolimus pre treatment blocks or reverses incubation-related changes in mTOR/Akt1 signaling within vmPFC subregions

Using the tissue obtained from experiment 1 rats, we also determined everolimus’ effects upon indices of mTOR/Akt1 signaling within the PL and IL and its temporal selectivity. No time-dependent changes were detected for the total protein expression of Akt1, P70S6K, or rpS6 within either subregion of VEH-pretreated rats (Supplementary Table 2) nor were any effects of everolimus detected upon total protein expression at either withdrawal time-point (Supplementary Table 2; Supplementary Fig. 2).

**Fig. 3:**
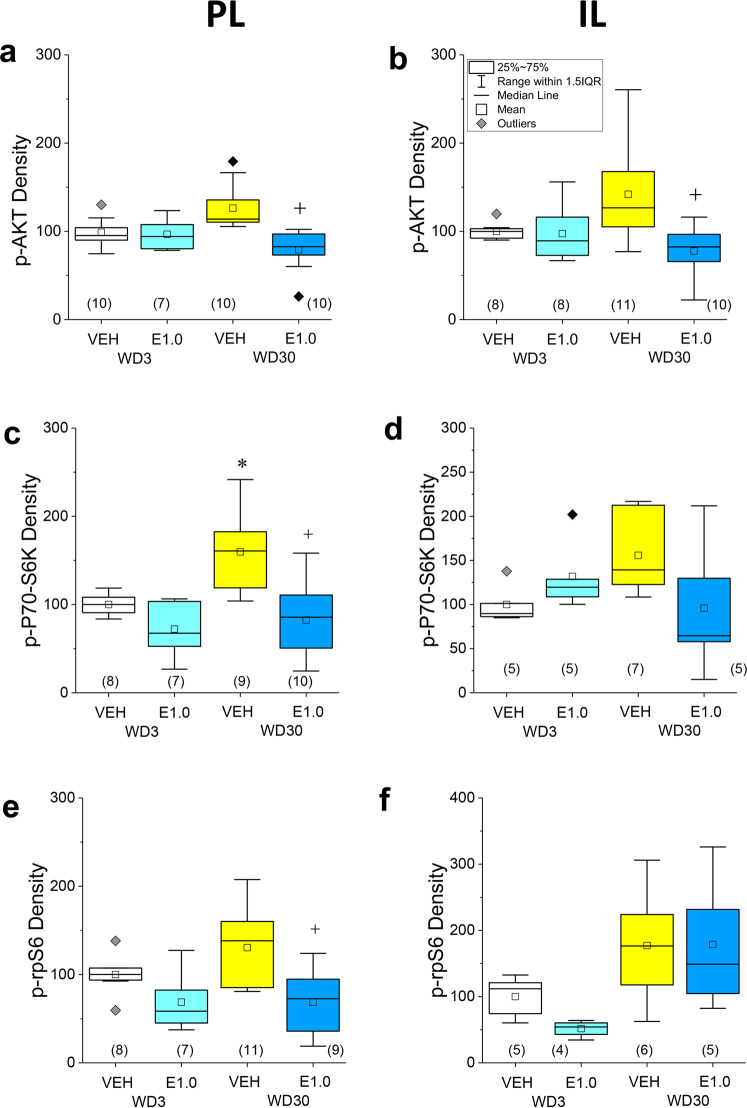
Acute everolimus pre treatment reverses incubation-related activation of Ak1/mTOR signaling in the PL and IL. The immunoblotting results for p(Ser473)-Akt1, p(Thr389)-P70S6K, and p(Ser234/235)-rpS6 expression within the PL are presented in the left panels, respectively from top to bottom. For direct comparison, the results for the IL are presented in the right panels. Relative to their VEH-pretreated controls, 1.0 mg/kg everolimus (E1.0) selectively reduced p(Ser473)-Akt1 within both the **a** PL and **b** IL of rats tested for incubated responding on WD30. **c** Relative to 3WD-VEH controls, p(Thr389)-P70S6K levels were significantly higher in the PL—an effect reduced by everolimus pre treatment. **d** In contrast, the group differences in p(Thr389)-P70S6K levels in the IL were not statistically significant. Relative to 3WD-VEH controls, the levels of p(Ser235/236)-rpS6 were not significantly elevated in either the PL (**e**) or IL (**f**) of 30WD-VEH rats, although everolimus reduced protein expression in rats tested on WD30. As indicated in **b**, the data are presented as box plots in which the mean is represented by, the median by —, outliers are indicated by ♦’s, the box represents the interquartile range (IQR) and the error bars represent 1.5 X IQR (see **d**). *p < 0.05 WD3-VEH vs. WD30-VEH (incubation); ^+^*p* < 0.05 30WD-VEH vs. 30WD-E1.0 (Everolimus effect) as determined by Tukey–Kramer comparisons corrected for multiple comparisons.

A time-dependent increase in p(Ser473)-Akt1 levels was observed within both the PL (Fig. 3a; *p* = 0.04) and the IL (Fig. 3b; *p* = 0.03) of VEH-pretreated rats, although this increase was not statistically significant when the analyses were corrected for multiple comparisons (*α* = 0.016). everolimus did not alter p(Ser473)-Akt1 expression within either subregion on WD3 (for PL, *p* = 0.70; for IL, *p* = 0.82), but significantly lowered phospho-protein levels within both subregions on WD30 (for PL, *p* = 0.0001; for IL, *p* = 0.002) (Fig. 3a, b).

A significant time-dependent increase in p(Thr389)-P70S6K expression was observed within the PL of VEH controls (Fig. 3c; *p* = 0.003), with a similar non-significant trend observed also in the IL (Fig. 3d; *p* = 0.03; *α* = 0.016). Everolimus pre treatment lowered the PL levels of p(Thr389)-P70SK6 at both withdrawal time-points, but the pretreatment effect was significant only on WD30 (Fig. 3c; for WD3, *p* = 0.02; for WD30, *p* = 0.001). No everolimus effect was detected for the expression of p(Thr389)-P70S6K in the IL (Fig. 3d; *p*’s > 0.11).

The time-dependent increase in expression of p(Ser235/236)-rpS6 exhibited by VEH controls was not statistically significant for both vmPFC subregions (Fig. 3e, f; for PL, *p* = 0.08; for IL, *p* = 0.1). Everolimus pre treatment lowered p(Ser235/236)-rpS6 within the PL, but again, this effect was statistically significant only at the WD30 time-point (Fig. 3e; for WD3, *p* = 0.04; for WD30, *p* = 0.003; *α* = 0.016), while a non-significant reduction in the IL expression of p(Ser235/236)-rpS6 was observed only in rats tested on WD3 (Fig. 3f; for WD3, *p* = 0.02; for WD30: *p* = 0.98; *α* = 0.016).

These immunoblotting data support the brain penetrance of everolimus and confirm that acute oral dosing with 1.0 mg/kg everolimus can reduce the activational state of Akt1, P70S6K, and rpS6 selectively within the PL of rats exhibiting incubated cocaine-seeking.

### Everolimus pre treatment reverses incubation-related changes in mGlu1/5 expression within the PL

**Fig. 4:**
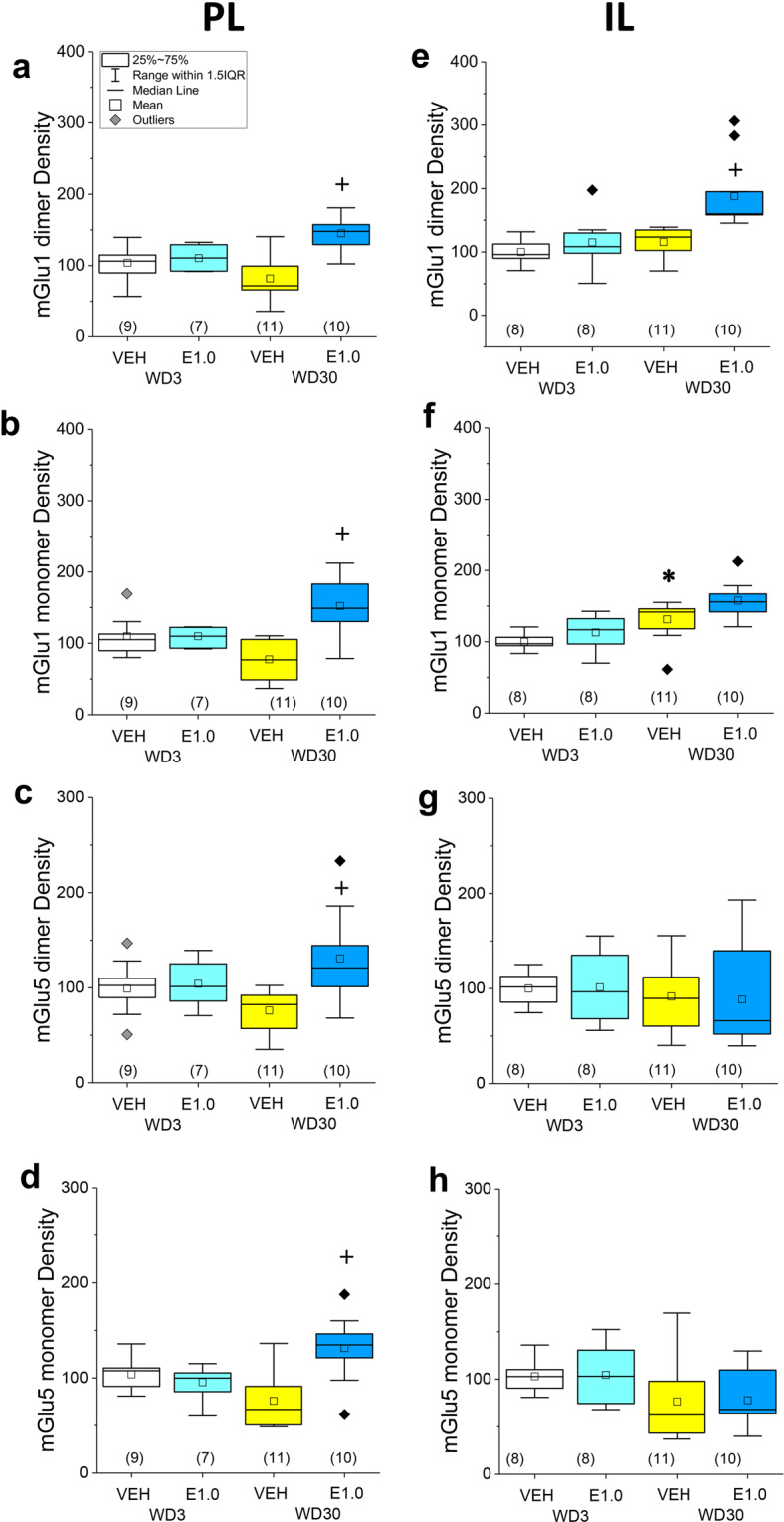
Everolimus pre treatment reverses incubation-related changes in mGlu1/5 expression within the PL. The immunoblotting results for the expression of the monomer and dimer forms of mGlu1 and mGlu5 within the PL are presented in the left panels. For direct comparison, the results for the IL are presented in the right panels. **a**–**d** In the PL, a non-significant reduction in the expression of both the dimer and monomer forms of each receptor was observed in 30WD-VEH vs. 3WD-VEH controls. Relative to 30WD-VEH rats, everolimus increased mGlu1/5 expression in rats tested on WD30. **e**–**h** Relative to 3WD-VEH controls, 30WD-VEH rats exhibited a significant increase in the IL levels of the mGlu1 monomer only. Everolimus significantly elevated IL levels of the mGlu1 dimer in rats tested on 30WD, with no other significant changes in protein expression detected in this subregion. As indicated in **a**, the data are presented as box plots in which the mean is represented by, the median by —, outliers are indicated by ♦’s, the box represents the interquartile range (IQR) and the error bars represent 1.5 X IQR (see **d**). *p < 0.05 WD3-VEH vs. WD30-VEH (incubation); ^+^*p* < 0.05 30WD-VEH vs. 30WD-E1.0 (Everolimus effect) as determined by Tukey–Kramer comparisons corrected for multiple comparisons.

VEH-pretreated rats exhibited a time-dependent, but not statistically significant, reduction in the PL levels of the monomer form of mGlu1 (Fig. 4b; *p* = 0.02; *α* = 0.016), with no change detected for the dimer form of this receptor (Fig. 4a; *p* = 0.12). Time-dependent reductions in both the dimer and monomer forms of mGlu5 were also observed within the PL for VEH-pretreated rats, but neither adaptation reached statistical significance (Fig. 4c, d; for dimer, *p* = 0.048; for monomer, *p* = 0.01; *α* = 0.016). In contrast, mGlu1 monomer expression within the IL of VEH-pretreated rats increased significantly as a function of withdrawal (Fig. 4f; *p* = 0.006), while no other time-dependent receptor changes were detected (for mGlu1 dimer, *p* = 0.13, Fig. 4e; for mGlu5 dimer, *p* = 0.56, Fig. 4g; for mGlu5 monomer, *p* = 0.15, Fig. 4h).

Although everolimus pre treatment did not affect PL levels of either mGlu1 or mGlu5 on WD3 (*p*’s > 0.30; see Supplementary Table 2), pretreatment elevated both the monomer and dimer forms of mGlu1 and mGlu5 on WD30 (Fig. 4a–d; *p*’s ≤ 0.0001; see Supplementary Table 2). With the exception of an increase in mGlu1 dimer expression on WD30 (Fig. 4e, *p* = 0.001), no everolimus effects were detect for the expression of these receptors within IL (Fig. 4f–h; *p*’s > 0.45; see Supplementary Table 2). These immunoblotting data indicate that the incubated cocaine-craving observed under the present self-administration procedures is associated with a strong trend towards a PL-selective reduction in mGlu1 and mGlu5 expression and that acute, oral, dosing with 1.0 mg/kg everolimus is sufficient to reverse these neuroadaptations within the PL and to augment mGlu1 dimer expression also within the IL.

### Everolimus pre treatment reverses incubation-related changes in Homer expression within the PL

**Fig. 5:**
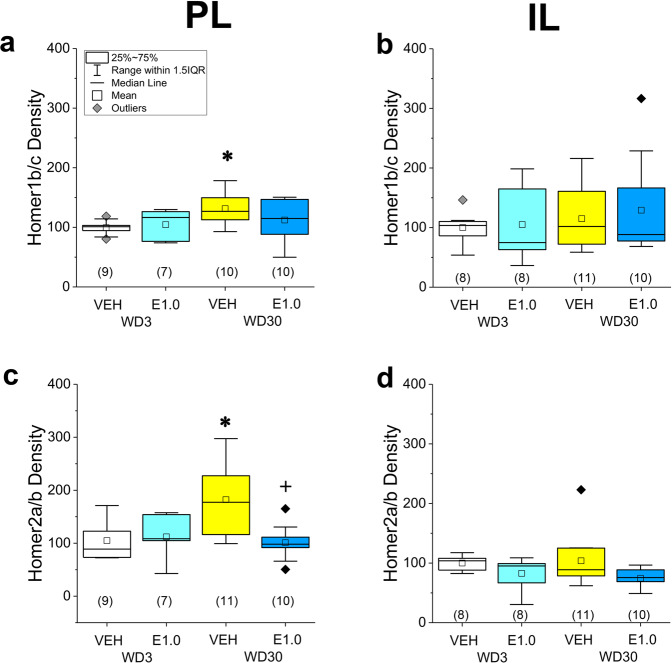
Everolimus pre treatment reverses incubation-related changes in Homer expression within the PL. The immunoblotting results for the expression of Homer1b/c and Homer2a/b within the PL are presented in the left panels. For direct comparison, the results for the IL are presented in the right panels. PL expression of both Homer1b/c (**a**) and Homer2a/b (**b**) increased as a function of withdrawal in vehicle (VEH)-pretreated rats, and everolimus lowered the levels of Homer2a/b on WD30. No group differences were detected for Homer1b/c (**c**) or Homer2a/b (**d**) expression within the IL. As indicated in **a**, the data are presented as box plots in which the mean is represented by, the median by—, outliers are indicated by ♦’s, the box represents the interquartile range (IQR) and the error bars represent 1.5 X IQR. *p < 0.05 WD3-VEH vs. WD30-VEH (incubation); ^+^*p* < 0.05 30WD-VEH vs. 30WD-E1.0 (Everolimus effect) as determined by Tukey–Kramer comparisons corrected for multiple comparisons.

Time-dependent increases in both Homer1b/c (Fig. 5a; *p* = 0.001) and Homer2a/b (Fig. 5b; *p* = 0.005) were observed within the PL of VEH-pretreated rats, with no changes detected within the IL (Fig. 5c, d; *p*’s > 0.40, see Supplementary Table 2). On WD3, everolimus pre treatment did not alter Homer1 and Homer2 expression within either the PL (*p*’s > 0.55) or the IL (*p*’s > 0.06; see Supplementary Table 2). In contrast, pretreatment on WD30 significantly lowered Homer2a/b expression within the PL (Fig. 5b; *p* = 0.002), with a non-significant reduction detected also for Homer1b/c within the PL (Fig. 5a; *p* = 0.09) and for Homer2a/b within the IL (Fig. 5d; *p* = 0.06). No everolimus effect was detected for IL expression of Homer1/c on WD30 (Fig. 5c; *p* = 0.64). These immunoblotting results for Homer proteins indicate that incubated cocaine-seeking is associated with elevated Homer1b/c and Homer2a/b expression selectively within the PL subregion of the vmPFC, the latter of which is reversed by acute, oral, dosing with everolimus.

### Protein correlates of cocaine-seeking within vmPFC subregions

When the entire vmPFC is examined, cue-elicited cocaine-seeking is positively correlated with indices of PI3K/Akt1 signaling [4] and Homer2 expression [25], but inversely correlated with the expression of the monomer forms of mGlu1 and mGlu5 [3]. To determine the subregional selectivity of these correlates of cocaine-seeking, comparable correlational analyses were performed on the PL and IL tissue from the rats in experiment 1. As detailed in the Supplementary information, we replicated a predictive relationship between cocaine-seeking and indices of mTOR/Akt1 activation within both the PL and IL (Supplementary Fig. 4). Homer2 expression in the PL also predicted drug-seeking (Supplementary Fig. 6), while an inverse relationship between cocaine-seeking and mGlu1 and mGlu5 monomer and dimer expression was detected in the PL only (Supplementary Fig. 5). Thus, just as incubated cocaine-seeking generalizes across different self-administration procedures, so too do their biochemical correlates.

## Discussion

Rat models of incubated cocaine-craving indicate an important role for PI3K/mTOR signaling within the PL-NAc projection in heightened drug-cue reactivity during protracted drug withdrawal [4, 14]. While intracranial drug delivery approaches facilitate understanding of the neural loci and molecular mechanisms involved in mediating a particular drug effect, such approaches are currently not experimentally, let alone clinically, feasible in humans. Further, such approaches cannot inform as to issues associated with drug bioavailability and provide much less insight into potential off-target and side-effects than do systemic routes of drug administration. Here, we examined the effects of systemic treatment with the FDA-approved allosteric mTOR inhibitor everolimus upon cue-elicited cocaine-craving during early and protracted withdrawal. Consistent with a significant body of clinical and preclinical literature [22, 23, 27–36], our findings support the oral bioavailability, brain penetrance, and relative safety of everolimus by demonstrating that acute, oral, dosing reduces cue-elicited craving only in rats exhibiting incubated cocaine-seeking as well as indices of mTOR pathway activation within the vmPFC. When administered at doses equal to, or below, those employed in rodent models of other disease [22, 23, 27–36], everolimus dose-dependently reduced incubated cocaine-craving for at least a 24-h period, which is a finding that may relate to its relatively long terminal half-life of ~50 h [32]. Oral administration of 1.0 mg/kg everolimus did not affect responding on the inactive lever in cocaine-experienced rats and the maximum dose did not impact cue-elicited sucrose-craving or cocaine-craving during early withdrawal. Thus, we conclude intact mTOR/P70S6K/rpS6 function is not required for cue-elicited drug-craving behavior per se, but is critical for its intensification during protracted withdrawal. While it remains to be determined [1] whether everolimus might reduce cue- or drug-elicited responding in other models of cocaine-taking or -craving and [2] how long the “anti-incubation” effect persists, the present data argue that acute, oral dosing with low-dose everolimus can produce a relatively long-lasting reduction in cocaine cue reactivity during protracted withdrawal, with no obvious off-target motivational or motor effects in either cocaine-naïve or -experienced subjects.

### Everolimus blocks incubated cocaine-craving, concomitant with reduced expression of phosphorylated Akt1, P70S6K, and rpS6 within the PL

In both the appetitive and aversive domains, a dorsal-ventral distinction is reported with respect to how vmPFC regulates the expression of conditioned behavior [41–49]. Extending prior immunoblotting results for the entire vmPFC of rats with a 10-day history of 6 h-access to IV cocaine [4], we detected evidence of increased indices of PI3K/Akt1/mTOR activation within both subregions of rats expressing incubated cocaine-seeking, with the most robust effect observed for p(Thr387)-P70S6K within the PL. Admittedly, the magnitude of the incubation-related increase in p(Ser473)-Akt1 expression within both the PL and IL of the rats in the present study was less than that reported in our previous report [4]. This likely reflects the “hybrid” self-administration procedure employed here, in which rats were trained to self-administer cocaine for 6 h on the first day of training and for 2 h/day for the remaining 9 days of self-administration. Collectively, the results of these two studies suggest that while the magnitude of vmPFC Akt1 activation may vary as a function of total cocaine intake in rats, the direction of the effect generalizes across intake histories and strengthens their relevance for the human condition, in which the duration and patterning of drug-taking history is highly variable. Further, the “anti-incubation” effects of everolimus were associated with a blockade of this increased PI3K/Akt1/mTOR signaling activation. Although we did not assay for everolimus’ effects in cocaine-naïve subjects, it is notable that everolimus did not affect p(Ser473)-Akt1 expression in rats tested in early cocaine withdrawal. In line with this observation, site-directed inhibition of PI3K/Akt1 signaling within the PL is sufficient to block incubated cocaine-craving and to do so for at least a 24-h period [4]. Although the functional relevance of PI3K/Akt1 hyperactivity within the IL for incubated responding has not been vetted, inhibiting glutamate release within this subregion reduces incubated cocaine-craving to a similar extent as that produced by comparable manipulation of the PL [26]. Taken together, these findings argue time-dependent increases in PI3K/Akt1 activity within both vmPFC subregions as drivers of incubated drug-craving and pose systemic treatment with an allosteric mTOR inhibitor as a means by which to mitigate this excessive signaling and normalize drug-cue reactivity.

### Everolimus reverses incubation-related changes in Homer2 expression

When the entire vmPFC is considered, incubated cocaine-seeking is associated with near-doubling of the expression of the glutamate receptor scaffolding protein isoform Homer2a/b [25]. Here, this effect occurs selectively within the PL and again, it is notable that this molecular correlate of incubation generalizes between limited and excessive cocaine intake histories. Aligning with our earlier study [25], Homer1b/c expression was less sensitive to cocaine withdrawal as we detected a relatively small (<50%) increase in protein expression within the PL during the incubation test. Homer proteins interact with the long isoform of the GTP-ase PI3K Enhancer (PIKE) to activate PI3K-dependent signaling [50] leading to the phosphorylation of Akt1 on Ser473 [17–19]. Thus, it is tempting to speculate that increased Homer [2]-PIKE scaffolding may contribute to the heightened Akt1 phosphorylation detected in rats expressing incubated cocaine-seeking. Indeed, Homer scaffolding to mGlu5 is required for receptor-mediated activation of PI3K/Akt1/mTOR signaling in brain [51]. Although Homer1 and Homer2 levels did not vary within the IL as a function of cocaine withdrawal, everolimus reduced Homer2 expression within both subregions of rats exhibiting incubated responding, which might contribute to inhibitor effects upon Akt1 phosphorylation in both subregions. However, arguing against a Homer-PIKE-PI3K/Akt1 mechanism as the sole driver of incubated craving, virus-mediated knockdown of Homer2 within the PL does not impact the magnitude of responding during the incubation test nor does it affect cue-induced reinstatement of cocaine-seeking following extinction [25]. It remains to be determined if interrupting Homer2-PIKE interactions within both the PL and the IL subregions (or selectively within the IL) would exert a more pronounced effect upon cue-elicited craving.

### Everolimus may facilitate the consolidation of extinction learning and increases mGlu1 and mGlu5 expression

As highlighted in several reviews [48, 49, 52, 53], there is little debate in the clinical literature that drug-cue hyper-reactivity is a major cordon to addiction recovery, with recommendations for therapeutics promoting extinction learning as a means to inhibit pathogenic memories in substance use disorder and reduce relapse vulnerability. We [25] and others [54] have demonstrated that cocaine-experienced rats exhibit intact (within-session) extinction learning when re-exposed to the drug-taking context and allowed to respond for discrete cocaine-associated cues during a test for incubated responding. Such observations, in the face of no apparent between-session extinction (3–5; present study) argue that the neural mechanisms governing the acquisition of extinction learning are functional in rats exhibiting incubated cocaine-craving, whereas those governing the consolidation of that extinction learning and/or its retrieval are impaired [6]. Consistent with the putative cognitive-enhancing potential of mTOR inhibitors [19, 26, 31–33], everolimus administration, either prior to, or immediately following, the first drug-cue re-exposure session, reduces cue-reactivity the next day. While the within-subject design of the posttreatment manipulation in experiment 2 limits interpretation, the fact that everolimus post treatment was effective at reducing subsequent cue-elicited responding nevertheless aligns with the interpretation that acute oral everolimus treatment may reduce drug-cue hyper-reactivity, at least in part, by facilitating the consolidation of extinction learning during initial drug-cue re-exposure. To further this “consolidation hypothesis”, it will be important to determine the critical period for extinction learning consolidation in drug-incubated rats and then compare the relative efficacy of everolimus post treatment inside and outside this window for reducing cue-elicited responding. Given the relatively long terminal half-life of everolimus [32], it will also be important to relate its enduring effect on behavior to its presence in the brain and examine for behavioral effects at a time when the drug is completely cleared from the body.

Positron emission tomography studies reveal a reduction in mGlu5 radioligand binding throughout the mesocorticolimbic system in cocaine use disorder [55, 56]. Akin to our animal model of incubated cocaine-craving [3], this mGlu5 deficit worsens in humans over the course of drug abstinence [57]. The consolidation of extinction learning requires intact mGlu1 and mGlu5 function within vmPFC [48, 52] and we have amassed both correlative and causal evidence supporting reduced mGlu1 and mGlu5 expression within vmPFC as key to maintaining the persistently high levels of cue reactivity exhibited by rats during a test for incubation [3]. Here, both the monomer and surface-expressed dimer forms of mGlu1 and mGlu5 are reduced within the PL of rats expressing incubated responding. Further, acute everolimus pre treatment augmented mGlu1 and mGlu5 expression in the PL and mGlu1 expression within the IL of rats exhibiting incubated responding above that expressed by rats tested in early withdrawal and both everolimus pre- and post-treatment reduced subsequent cue-reactivity in rats tested on WD30.

Given the putative role for mTOR signaling in activating protein translation (c.f., [58, 59]), it is not clear at present if and how mTOR inhibition directly relates to increased mGlu1/5 expression within PFC subregions. However, mTOR is reported to inhibit the activity of the translational repressor fragile X mental retardation protein [60, 61], which normally stalls the translation of mGlu5 (as well as other important postsynaptic scaffolding proteins to include PIKE) [59]. This raises the intriguing possibility that everolimus may rescue what appears to be a difficulty in extinction learning/memory by relieving translational repression on mGlu1/5. Alternatively, mGlu1/5 receptors undergo rapid phosphorylation-dependent desensitization upon their activation [61]. While we did not examine the phosphorylation status of mGlu1/5, the complexity of the mGlu5 interactome, which includes mTOR [62, 63], raises the alternate possibility that everolimus attenuate subsequent cue-reactivity by blunting kinase-dependent receptor down-regulation. The precise mechanism(s) through which acute everolimus blocks incubated cocaine-craving require further investigation. Equally, it will be important to determine whether or not the present findings generalize to female subjects (whom exhibit similar incubation but also estrous cycle modulation of drug-seeking behavior independent of cocaine intake histories [64, 65], to rats of different ages, to other models of cocaine use disorder, particularly those that appear to better model the addictive state, as well as to other drugs of abuse. Nevertheless, the present data indicate the potential for repurposing current FDA-approved PI3K/Akt1/mTOR inhibitors for craving reduction during protracted recovery in Cocaine Use Disorder and provide a foundation for preclinical investigation of how PI3K/Akt1/mTOR signaling gates behavioral reactivity to drug-associated cues.

## Funding and disclosure

Funding for this project was provided in part by NIH grant AA024044 (KKS), as well as funds from the Academic Senate (KKS), the Faculty Research Assistance Program (KKS) and the Undergraduate Research and Creative Activities Program the University of California Santa Barbara (ASC, MCK, ANF). BDB was supported by a UC LEADS Scholarship and CLJC was supported by an NSF Graduate Fellowship. The authors declare no competing interests.

## Supplementary information

Supplemental Materials
