# Supplementary material for: Preclinical evidence to support repurposing everolimus for craving reduction during protracted drug withdrawal
Source: Neuropsychopharmacology. 2021 Jun 29;46(12):2090–100. doi: 10.1038/s41386-021-01064-9 (PMC8505628; doi:10.1038/s41386-021-01064-9)

SUPPLEMENT

SUPPLEMENTAL MATERIALS AND METHODS

Subjects. Rats were housed in pairs in a distinct colony room in polyethylene cages under a reverse 12-h light/dark cycle (lights off: 7:00 AM). Food and water were available *ad libitum* throughout the course of the study. Rats were housed in pairs in a distinct colony room in polyethylene cages under a reverse 12-h light/dark cycle (lights off: 7:00 AM). Food and water were available *ad libitum* throughout the course of the study.

Surgical Procedures. Under ketamine/xylazine anesthesia (respectively 56.25 and 7.5 mg/kg, IM; 2 mg/kg banamine analgesic, SC, for post-operative pain), animals slated for cocaine self-administration studies were implanted with a chronic indwelling jugular catheter (13 cm long; 0.3 mm inner diameter, 0.64 mm outer diameter; Dow Corning) into the right jugular vein, as described previously by our group (e.g., 1-3). Each catheter ran subcutaneously around the shoulder to the back where it was secured to a threaded 22-gauge metal guide cannula (Plastics One), which emerged from the midline of the animal's back perpendicular to the dorsal surface. An obturator covered the open end of the cannula to protect from contamination and the cannula was held in place via a small swatch of Bard Mesh (C. R. Bard) to which it was cemented. The mesh was, in turn, laid flat subcutaneously on the animal's back. A minimum of 4 days was allowed for recovery, with jugular catheter patency maintained by daily flushing of sterile heparin/timentin/saline (60 IU/ml and 100 mg/ml, respectively; vol ¼ 0.1 ml) and confirmed weekly by intravenous infusion of 5 mg/kg brevital (JHP Pharmaceuticals, Parsippany, NJ, USA).

Cocaine and Sucrose Self-administration*.* Animals were trained to press the "active" lever for delivery of either intravenous cocaine (0.25 mg in 0.1 ml saline infusion; NIDA, Bethesda, MD) or a 45 mg banana-flavored sucrose pellet (Bio Serv; Flemington, NJ, USA) into a food hopper, under an FR1 schedule of reinforcement. In both cases, the delivery of either reinforcer was signaled by a 20-s light-tone compound stimulus, during which time responding resulted in no programmed consequences (i.e., time-out) and depression of the "inactive lever" had no programmed consequences at any time during testing.

Rats were then left undisturbed in their home cages for either 3 or 30-46 days, at which time we tested the effects of oral Everolimus upon cue-elicited responding. The range of later withdrawal times reflected technical issues that postponed the testing of cocaine self-administering animals in one cohort (n=3 for VEH and n=4 for 1 mg/ml Everolimus), but is consistent with the ranges employed by other laboratories investigating the neurobiology of incubated cocaine-craving (e.g., 5-8). The average number of reinforcers earned was determined over the last 3 days of self-administration and the animals were assigned to one of several drug pretreatment groups, ensuring similar levels of cocaine/sucrose self-administration across the different conditions.

Gavage Infusion Procedures. During the 2 days prior to testing for cue-elicited responding, rats were habituated, once daily, to the handling and gavage infusion procedures required for oral drug delivery. For this, rats were lightly restrained and a plastic disposable gavage tube (early withdrawal: 18GA x 50mm; later withdrawal: 15GA x 100mm; Instech, Plymouth Meeting, PA, USA) was inserted, followed by infusion of water (vol: 1 ml/kg). For all rats, the tests for cue-elicited drug/sucrose-seeking were conducted in the same operant chamber as that employed during the self-administration phase of the study and were 2-h long, as in prior immunoblotting work (1). During these cue tests, depression of the formerly reinforced, "active", lever resulted in the presentation of the 20-sec tone/light stimulus, while depression of the inactive lever had no programmed consequences.

Immunoblotting. As some evidence indicates that Everolimus has relatively low brain penetrance versus other rapamycin derivatives (9-12), we first immunoblotted for indices of PI3K/Akt1/mTOR pathway activation within vmPFC subregions by examining the levels of p(Ser473)-Akt1 (13-15) [which is elevated within the vmPFC of cocaine-incubated rats (1)], as well as p(Thr389)-P70 rpS6kinase (**P70S6K**) (16,17). P70S6K phosphorylates serines 235/236 of rpS6 (18) [the latter of which is upregulated within the nucleus accumbens of cocaine-incubated rats (7)]. We successfully immunoblotted for all of these phospho-proteins and their non-phosphorylated forms in the PL tissue. However, despite our best efforts (and the ability to detect our other proteins of interest), we encountered issues with reliable immunodetection of P70S6K, rpS6 and their phosphorylated forms in the IL tissue from approximately half of our rats. As such, the sample sizes employed in the statistical analyses of these proteins is lower than those employed for the other proteins of interest examined in the IL.

The immunoblotting procedures employed herein to examine for mGlu1 and mGlu5 (e.g., 1,19,20), Homer1b/c and Homer2a/b (19-21), Akt1 (3,20), rpS6 and P70S6K (22) were similar to those described previously by our group and the details regarding the preparation of tissue homogenates, electrophoresis and protein transfer are provided in these prior reports. The following rabbit primary antibodies were used: Homer2a/b (1:1000 dilution; Synaptic Systems; 160 203), mGlu5 (1:1000 dilution; Millipore; AB5675), P70S6 (1:250 dilution; Cell Signaling Technology, 9202), rpS6 (1:250 dilution; Cell Signaling Technology; 2217), and AKT (1:1000 dilution; Cell Signaling Technology; 9272). The following mouse primary antibodies were also employed: Homer1b/c (1:1000 dilution; Santa Cruz Biotechnology; sc-25271), mGlu1 (1:1000 dilution; BD Biosciences; 610965), p(Thr389)-P70S6K (1:250 dilution; Cell Signaling Technology; 9206), p(Ser235/236)-rpS6 (1:250 dilution; Cell Signaling Technology; 62016); p(Ser473)-Akt1 (1:1000 dilution; Cell Signaling Technology; 4051). A rabbit primary anti-calnexin antibody (1:1000 dilution; Enzo Life Sciences; ADI-SPA-860) was used as a control to ensure even protein loading and transfer. Membranes were subsequently washed with PBST, incubated in either a goat anti-rabbit IRDye 800 CW secondary antibody (1:10,000 dilution; Li-Cor; 925-3221) or a goat anti-mouse IRDye 680RD secondary antibody (1:10,000 dilution; Li-Cor; 925-68070), dried, and imaged on an Odyssey Infrared Imaging System (Li-Cor Biosciences, Lincoln, NE, USA). Raw values for each band were measured, and first normalized to their corresponding calnexin signal and then to the average value of the vehicle control (i.e., 3WD-VEH). Subsequently, the ratio of phospho-protein to total protein was obtained for each measurement. Examples of the resultant immunoblots are provided in the Supplemental Materials (**Suppl. Fig.3**). Note that for antibodies resulting in a doublet [e.g., P70S6K and p(Thr389)-P70S6K], both bands were included in the densitometric analysis. Signals with an abnormal dot-shape (e.g., lane 2 for Akt, p(Ser473)-Akt1 and Homer1b/c in **Suppl. Fig.3** were not included in the statistical analyses of the results.

SUPPLEMENTAL RESULTS

Experiment 1: Cocaine Self-Administration Behavior. To facilitate learning, the rats underwent a 6-h session on the first day of self-administration training, during which the rats exhibited cocaine-directed responding as evidenced by a nearly 7-fold difference in the number of presses on the ‘active’ versus ‘inactive’ lever (75.05 ± 13.46 vs. 11.16 ± 2.72), with rats receiving an average of 35.79 ± 4.24 cocaine infusions during this initial day of training. For the next 9 days, rats underwent daily 2-h cocaine self-administration sessions and the average number of active and inactive lever-presses, as well as cocaine infusions, over the last 3 days of training were, respectively: 32.69 ± 2.89, 2.33 ± 0.24, 25.76 ± 1.81.

Protein correlates of cocaine-seeking within vmPFC subregions. Indices of PI3K/Akt1 signaling within the entire vmPFC are correlated with cue-elicited cocaine-seeking (PI3K) (Szum). Here, we also observed a predictive relationship between cocaine-seeking and indices of mTOR/Akt1 activation within the PL of vehicle-pretreated rats, with the levels of p(Ser473)-Akt1, p(Thr389)-P70S6K, and p(Ser234/235)-rpS6 positively correlated with the magnitude of the drug-seeking response (**Suppl.Fig.4a-c**). Interestingly, a similar predictive relationship was also observed for p(Ser473)-Akt1 within the IL (**Suppl.Fig.4d**), while a statistical trend was noted for IL levels of p(Thr389)-P70S6K (**Suppl.Fig.4e**). There was no overt relationship detected between cocaine-seeking behavior and IL levels of p(Ser235/236)-rpS6 (**Suppl.Fig.4f**). No correlations were found between responding and the total expression of these proteins within either subregion (**Suppl.Fig.1**).

In the PL, both the dimer and monomer forms of mGlu1 and mGlu5 were inversely correlated with drug-seeking behavior (**Suppl.Fig.5a-d**). In contrast to the PL, no relationships were detected between mGlu1 and mGlu5 expression within the IL and drug-seeking behavior (**Suppl.Fig.5e-h**).

Homer1b/c and Homer2a/b expression was positively correlated with drug-seeking behavior, although this relation was stronger for Homer2a/b (**Suppl.Fig.6a,b**). In contrast, no relationship was apparent for either Homer isoform within the IL (**Suppl.Fig.6c,d**)

SUPPLEMENT REFERENCES

1. Ben-Shahar O, Sacramento AD, Miller BW, Webb SM, Wroten MG, Silve, HE et al. Deficits in ventromedial prefrontal cortex Group1 metabotropic glutamate receptor function mediate resistance to extinction during protracted withdrawal from an extensive history of cocaine self-administration. J Neurosci. 2013;33:495-506.

2. Shin CB, Serchia M, Shahin J, Ruppert-Mejor M, Szumlinski KK. Incubation of cocaine-craving relates to glutamate over-flow within ventromedial prefrontal cortex. Neuropharmacology. 2016; 102:103-110.

3. Szumlinski KK, Ary AW, Shin CB, Wroten MG, Courson J, Miller BW et al. (2018) PI3K activation within ventromedial prefrontal cortex regulates the expression of drug-seeking in two rodent species Addict Biol. 2018; 24:1216-1226.

4. Kerstetter KA, Aguilar VR, Parrish AB, Kippin TE. Protracted time-dependent increases in cocaine-seeking behavior during cocaine withdrawal in female relative to male rats. Psychopharmacology. 2008; 198:63-75.

5. Ma YY, Lee BR, Wang X, Guo C, Liu L, Cui R, et al. Bidirectional modulation of incubation of cocaine craving by silent synapse-based remodeling of prefrontal cortex to accumbens projections. Neuron. 2014; 83:1453-1467.

6. Tran-Nguyen LT, Fuchs RA, Coffey GP, Baker DA, O'Dell LE, Neisewander JL. Time-dependent changes in cocaine-seeking behavior and extracellular dopamine levels in the amygdala during cocaine withdrawal. Neuropsychopharmacology. 1998; 19:48-59.

7. Scheyer AF, Wolf ME, Tseng KY. A protein synthesis-dependent mechanism sustains calcium-permeable AMPA receptor transmission in nucleus accumbens synapses during withdrawal from cocaine self-administration. J Neurosci. 2014;34:3095–3100.

8. Werner CT, Stefanik MT, Milovanovic M, Caccamise A, Wolf ME. Protein Translation in the Nucleus Accumbens Is Dysregulated during Cocaine Withdrawal and Required for Expression of Incubation of Cocaine Craving. J Neurosci. 2018; 38:2683-2697.

9. Moes DJ, Guchelaar HJ, de Fijter JW. Sirolimus and everolimus in kidney transplantation. Drug Discov Today 2015; 20:1243-1249.

10. Asati V, Mahapatra DK, Bharti SK. PI3K/Akt/mTOR and Ras/Raf/MEK/ERK signaling pathways inhibitors as anticancer agents: Structural and pharmacological perspectives. Eur J Med Chem. 2016; 109:314-341

11. O'Reilly T, McSheehy PM, Kawai R, Kretz O, McMahon L, Brueggen J, et al. Comparative pharmacokinetics of RAD001 (everolimus) in normal and tumor-bearing rodents. Cancer Chemother Pharmacol. 2010; 65: 625-639.

12. Magaway C, Kim E, Jacinto E. Targeting mTOR and Metabolism in Cancer: Lessons and Innovations. Cells. 2019; 8: pii: E1584.

13. Ruggero D, Sonenberg N. The akt of translational control. Oncogene. 2005; 24:7426–7434.

14. King D, Yeomanson D, Bryant HE. PI3King the lock: targeting the PI3K/Akt/mTOR pathway as a novel therapeutic strategy in neuroblastoma. J Pediatr Hematol Oncol. 2015; 37: 245-251.

15. Sarbassov DD, Guertin DA, Ali SM, Sabatini DM. Phosphorylation and regulation of Akt/PKB by the rictor-mTOR complex. Science. 2005; 307:1098-1101.

16. Wullschleger S, Loewith R, Hall MN. TOR signaling in growth and metabolism. Cell .2006;124:471-84.

17. Hay N, Sonenberg N. Upstream and downstream of mTOR. Genes Dev. 2004; 18: 1926–1945.

18. Magnuson B, Ekim B, Fingar DC. Regulation and function of ribosomal protein S6 kinase (S6K) within mTOR signalling networks. Biochem J. 2012; 441:1-21.

19. Ary AW, Lominac KD, Wroten MG, Williams AR, Campbell RR, Ben-Shahar O, et al. Imbalances in prefrontal cortex CC-Homer1 versus –Homer2 expression promote cocaine-seeking behavior. J Neurosci. 2013; 33:8101-8113

20. Cozzoli DK, Courson J, Caruana AL, Miller BW, Thompson AB, Wroten M et al. Accumbens shell metabotropic glutamate receptor 5-associated signaling regulates binge alcohol drinking: Evidence from Drinking-in-the-Dark studies. Alcohol Clin Exp Res. 2012; 36: 1623-1633.

21. Gould AT, Sacramento AD, Wroten MG, Miller BW, Klugmann M, Ben-Shahar O et al. Extended access to intravenous cocaine imbalances ventromedial prefrontal cortex Homer1 versus Homer2 expression: Implications for relapse. Addiction Biol. 2015; 20: 148-157

22. Bailey J, Ma D, Szumlinski KK. Rapamycin attenuates the expression of cocaine-induced place-preference and behavioral sensitization. Addiction Biol. 2012; 17: 248-258.

SUPPLEMENTAL FIGURE LEGENDS

<u>Supplemental Figure 1:</u> Acute Everolimus pretreatment does not alter responding on the inactive lever in cocaine- or sucrose-experienced rats.
Inset: Summary of the procedural time-line of Experiment 2. No group differences were detected in the number of inactive lever presses emitted on Test 1 ***(a)*** or Test 2 ***(b)*** in Experiment 2 of incubated cocaine-seeking. ***c.*** Likewise, Everolimus post-treatment did not alter inactive lever-responding on Test 3. Everolimus pretreatment also did not alter the number of inactive lever presses emitted on either Test 1 ***(d)*** or Test 2 ***(e)*** in our study of sucrose-seeking. As summarized in Panel a, the data are presented as box plots in which the mean is represented by □, the median by —, outliers are indicated by ♦'s, the box represents the interquartile range (IQR) and the error bars represent 1.5 X IQR. The sample sizes are indicated in parentheses in their respective panels.

<u>Supplemental Figure 2:</u> Everolimus pretreatment has little to no effect upon the total protein expression of mTOR-related signaling molecules within vmPFC subregions.
The immunoblotting results for the total protein expression of Akt1, P70S6K, and rpS6 expression within the PL are presented in the left panels. For direct comparison, the results for the IL are presented in the right panels. Cartoons depicting the area of tissue dissection for each subregion are presented along the top. No group differences were detected for the total Akt (***a,b***), P70S6K (***c,d***) or rpS6 (***e,f).*** The data are presented as box plots in which the mean is represented by □, the median by —, outliers are indicated by ♦'s, the box represents the interquartile range (IQR) and the error bars represent 1.5 X IQR (see panel d'). Sample sizes are indicated in parentheses in each panel.

<u>Supplemental Figure 3:</u> Representative immunoblots for the various proteins examined in this report. ***Top:*** Entire immunoblots for the 90 kDa control protein calnexin within the PL (left) and IL (right), indicating the protein ladder obtained when electrophoresis is conducted using a Tris-Acetate (left) and Bis-Tris (left) gel. ***Bottom:*** Representative bands from comparable immunoblots conducted on either Tris-Acetate or Bis-Tris gels for the proteins examined in the PL (left) and the IL (right).

<u>Supplemental Figure 4:</u> Phospho-protein correlates of cocaine-seeking. The immunoblotting results for p(Ser473)-Akt1, p(Thr389)-P70S6K, and p(Ser234/235)-rpS6 expression within the PL are presented in the left panels. For direct comparison, the results for the IL are presented in the right panels. ***a-c,*** The levels of phospho-protein expression within the PL were all positively correlated with the magnitude of the drug-seeking response. ***d,*** The levels of p(Ser473)-Akt1 within the IL was also positively correlated with drug-seeking. ***e,*** A statistical trend was noted for IL levels of p(Thr389)-P70S6K, while ***f,*** no overt relationship was detected between cocaine-seeking behavior and IL levels of p(Ser235/236)-rpS6. For all panels, the results of the correlational analyses are presented in each panel, along with the total sample size of each experiment.

<u>Supplemental Figure 5:</u> Receptor correlates of cocaine-seeking. In the PL, both the dimer and monomer forms of mGlu1 and mGlu5 were inversely correlated with drug-seeking behavior (***a-d***). In contrast to the PL, no relationships were detected between mGlu1/5 expression within the IL and drug-seeking behavior (***e-h***). For all panels, the results of the correlational analyses are presented in each panel, along with the total sample size of each experiment.

<u>Supplemental Figure 6:</u> Homer scaffolding correlates of cocaine-seeking. Homer1b/c and Homer2a/b expression in the PL was positively correlated with drug-seeking behavior, although

this relation was stronger for Homer2a/b (***a,b***). In contrast, no relationship was apparent for either Homer isoform within the IL (***c,d***). For all panels, the results of the correlational analyses are presented in each panel, along with the total sample size of each experiment.

Supplemental Table 1: Comparison of the average data from the last 3 days of cocaine and sucrose self-administration for the different subgroups of rats employed in this study. Data represent the means ± SEMs of the number of rats indicated in parentheses. As detailed in the main text, statistical analyses failed to indicate any group differences in the number of active or inactive lever-presses or the number of cocaine/sucrose reinforcers earned prior to tested for Everolimus' effects.

**Dose-response Cocaine Study (Experiment 1)**					
	**WD3-VEH (n=9)**	**WD30-VEH (n=11)**	**WD30-E0.01 (n=7)**	**WD30-E0.1 (n=9)**	**WD30-E1.0 (n=7)**
**Active Lever-Presses**	25.19 ± 3.23	30.36 ± 5.04	47.07 ± 11.92	33.70 ± 6.44	30.33 ± 2.90
**Inactive Lever-Presses**	3.30 ± 0.71	1.42 ± 0.38	1.88 ± 0.43	2.58 ± 0.50	2.76 ± 0.50
**Reinforcers**	19.40 ± 1.54	26.36 ± 4.26	29.94 ± 6.37	26.88 ± 3.54	26.93 ± 2.71
**Sucrose Study**					
	**WD3-VEH (n=7)**	**WD30-VEH (n=8)**	**WD30-E1.0 (n=8)**		
**Active Lever-Presses**	150.71 ± 17.63	146.25 ± 16.15	120.13 ± 8.57		
**Inactive Lever-Presses**	5.86 ± 1.14	4.67 ± 0.54	5.79 ± 0.72		
**Reinforcers**	94.48 ± 8.29	90.13 ± 7.27	88.63 ± 5.00		
**Cocaine Replication Study**					
	**WD3-VEH (n=9)**	**WD3-E1.0 (n=7)**	**WD30-VEH (n=10)**	**WD30-E1.0 (n=10)**	
**Active Lever-Presses**	30.70 ± 2.17	35.33 ± 3.34	35.45 ± 1.18	35.90 ± 3.17	
**Inactive Lever-Presses**	2.48 ± 0.28	2.33 ± 0.38	2.98 ± 0.33	2.67 ± 0.22	
**Reinforcers**	28.44 ± 1.92	32.71 ± 3.41	32.90 ± 1.87	31.30 ± 2.41	

Supplemental Table 2: Statistical results of the *a priori* contrasts conducted on the immunoblotting data from vehicle (VEH)-pretreated rats on withdrawal day (WD) 3 versus 30 and between rats infused with VEH versus 1.0 mg/kg Everolimus (E1.0), separately on WD3 and WD30. Significant differences in protein expression are bolded. Abbreviations: PL=prelimbic cortex; IL=infralimbic cortex; t=total protein; p=phosphoprotein; P70S6K=P70 S6 kinase; rpS6=riboprotein S6.

	**VEH: WD3 vs. WD30**		**WD3: VEH vs. E1.0**		**WD30: VEH vs. E1.0**	
	**PL**	**IL**	**PL**	**IL**	**PL**	**IL**
**t-AKT**	p=0.70	p=0.10	p=0.49	p=0.84	p=0.24	p=0.72
**p-AKT**	**p=0.04**	**p=0.03**	p=0.70	p=0.82	**p=0.001**	**p=0.002**
**t-P70S6K**	p=0.39	p=0.52	p=0.07		p=0.09	
**p-P70S6K**	**p=0.003**	**p=0.03**	**p=0.02**	p=0.16	**p=0.001**	p=0.11
**t-rpS6**	p=0.75	p=0.21	p=0.85		p=0.34	
**p-rpS6**	p=0.08	p=0.1	**p=0.04**	**p=0.02**	**p=0.003**	p=0.98
**mGlu1 dimer**	p=0.12	p=0.13	p=0.58	p=0.37	**p<0.0001**	**p=0.01**
**mGlu1 monomer**	**p=0.02**	**p=0.006**	p=0.99	p=0.2	**p<0.0001**	p=0.48
**mGlu5 dimer**	**p=0.048**	p=0.56	p=0.72	p=0.93	**p=0.003**	p=0.87
**mGlu5 monomer**	**p=0.01**	p=0.15	p=0.35	p=0.72	**p=0.001**	p=0.94
**Homer1b/c**	**p=0.001**	p=0.44	p=0.58	p=0.84	p=0.09	p=0.64
**Homer2a/b**	**p=0.005**	p=0.81	p=0.72	p=0.07	**p=0.002**	p=0.06

Supplemental Fig.1

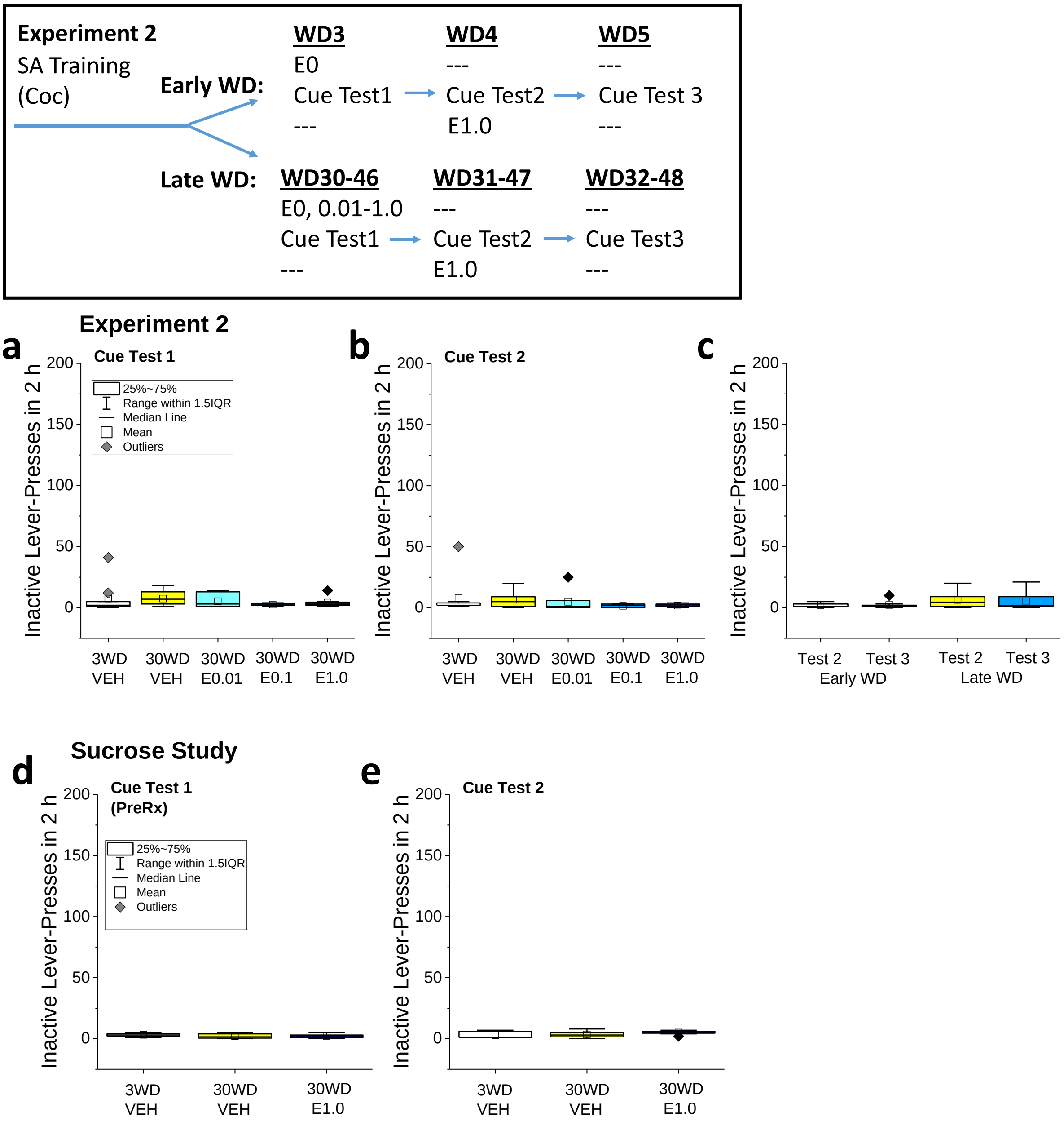

Supplemental Fig.2

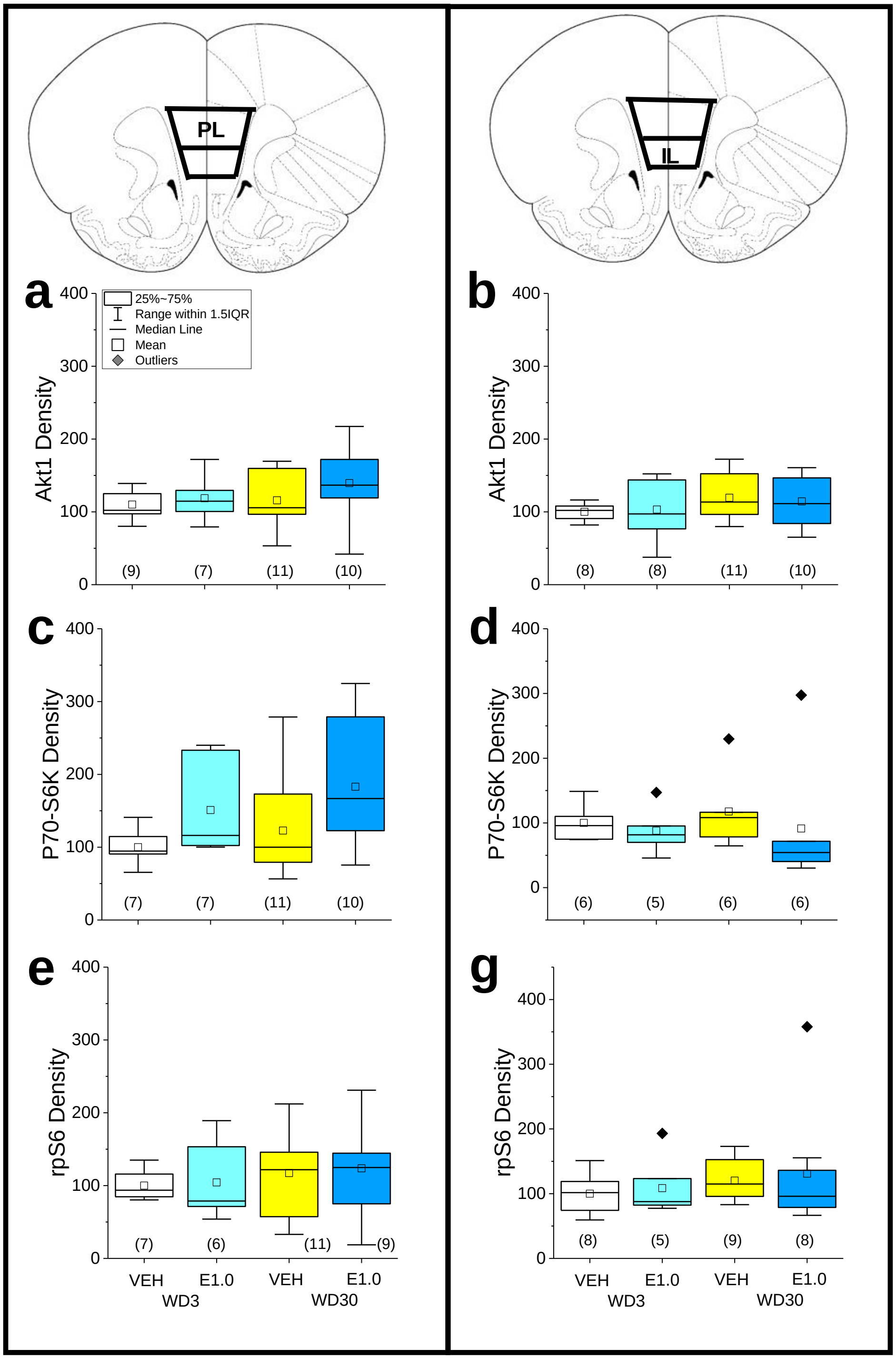

Supplemental Fig.3-representative immunoblots

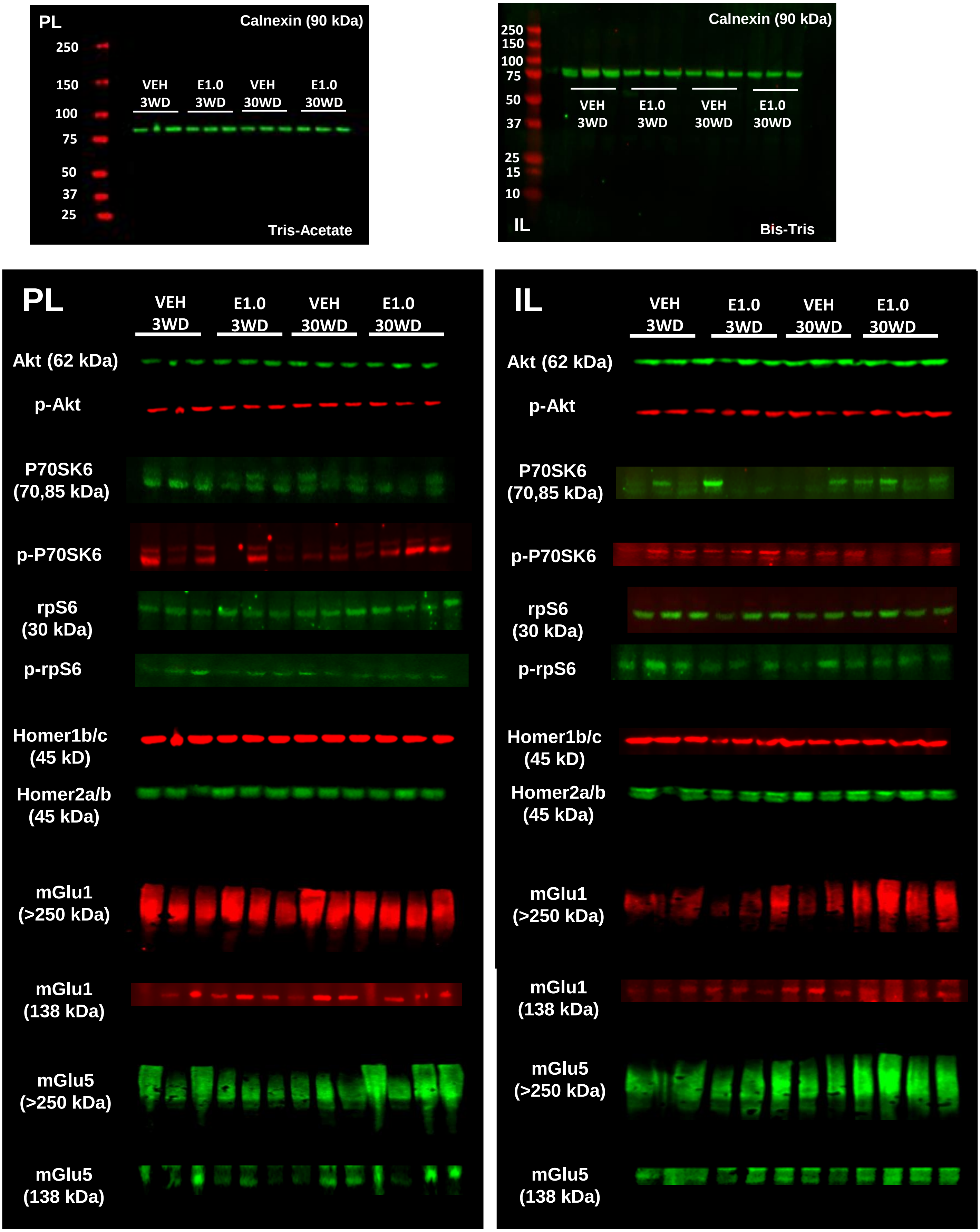

Supplemental Fig.4

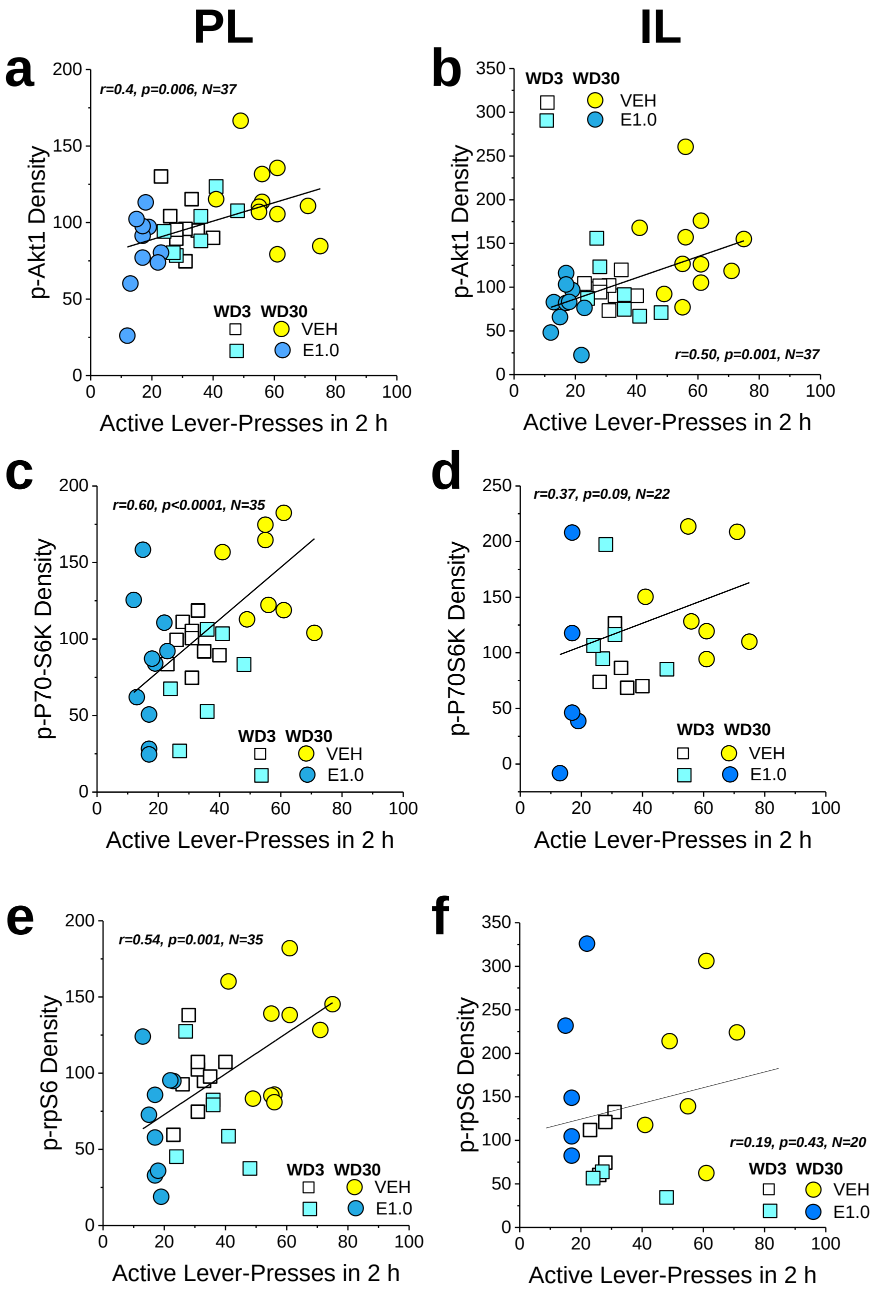

Supplemental Fig.5

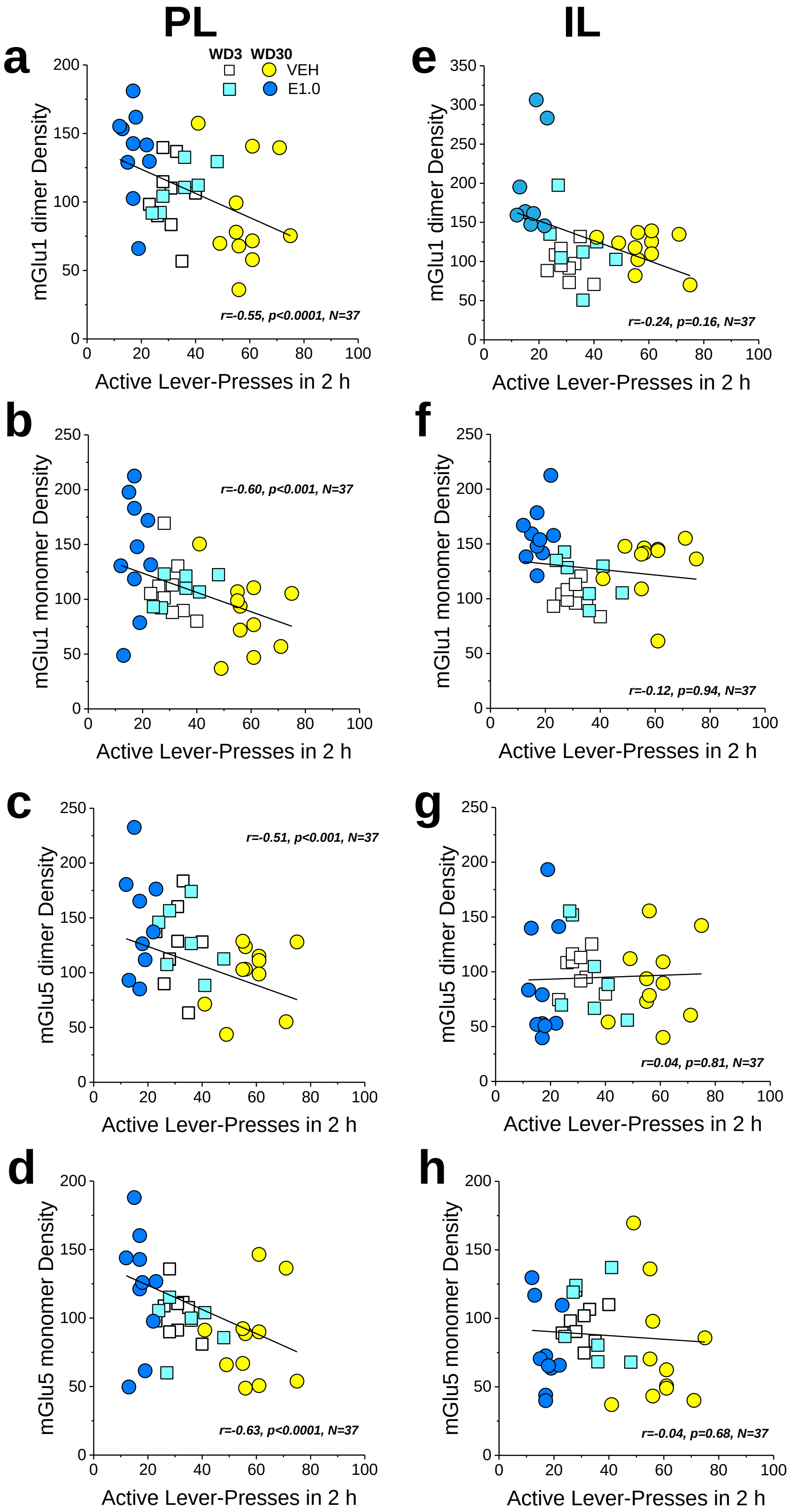

Supplemental Fig.6

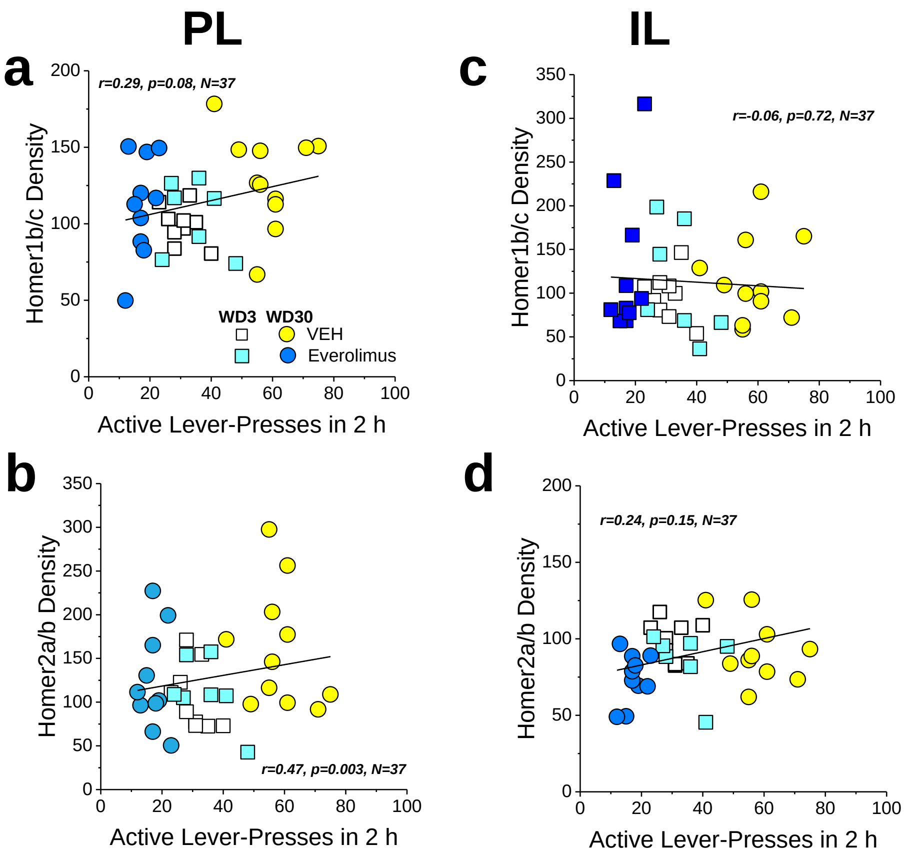

Supplement: Supplementary file 1 — Supplemental Materials [file 41386_2021_1064_MOESM1_ESM.pdf]
